# Recurrent Pediatric Thumb Carpometacarpal Joint Dislocation Due to Generalized Joint Laxity Successfully Treated With Ligament Reconstruction: A Case Report

**DOI:** 10.7759/cureus.65365

**Published:** 2024-07-25

**Authors:** Jun Tsujimoto, Takuya Uemura, Sadahiko Konishi, Hiroaki Nakamura

**Affiliations:** 1 Department of Orthopaedic Surgery, Osaka General Hospital of West Japan Railway Company, Osaka, JPN; 2 Department of Orthopaedic Surgery, Seikeikai Hospital, Sakai, JPN; 3 Department of Orthopaedic Surgery, Graduate School of Medicine, Osaka Metropolitan University, Osaka, JPN

**Keywords:** traumatic dislocation, thumb, joint laxity, child, trapeziometacarpal joint

## Abstract

Acute traumatic dislocation without fractures of the thumb carpometacarpal (CMC) joint is extremely rare in children. Treatment options, such as closed reduction with casting or pinning and open reduction with primary ligament repair, remain controversial. Here, we report the first case of an 11-year-old boy with recurrent left thumb CMC joint dislocation due to idiopathic generalized hyperjoint laxity, even after primary open reduction with capsular ligament repair of the thumb CMC joint, eventually treated with Eaton-Littler’s ligament reconstruction. Intraoperatively, a drill hole was made in the base of the first metacarpal bone while carefully preventing growth plate injury. Primary ligament reconstruction of the thumb CMC joint may be considered in pediatric cases with systemic hyperjoint laxity or recurrent thumb CMC joint dislocation. In such cases, Eaton-Littler’s ligament reconstruction is recommended for thumb CMC joint stability because two prime stabilizers of the dorsoradial ligament and the volar anterior oblique ligament (AOL) are appropriately reconstructed by a half-slip of the flexor carpi radialis tendon.

## Introduction

Acute traumatic isolated dislocation without fractures of the thumb carpometacarpal (CMC) joint is uncommon in adults and extremely rare in children [1,2]. The treatment approach for pediatric cases is still controversial, ranging from closed reduction with a cast or pinning to open reduction with primary ligament repair [3-7]. Moreover, recurrent cases after closed reduction and percutaneous pinning have also been reported [8,9]. Here, we report the first case of a recurrent pediatric thumb CMC joint dislocation due to idiopathic systemic joint laxity, even after open reduction with primary ligament repair, successfully treated with secondary ligament reconstruction.

## Case presentation

**Figure 1:**
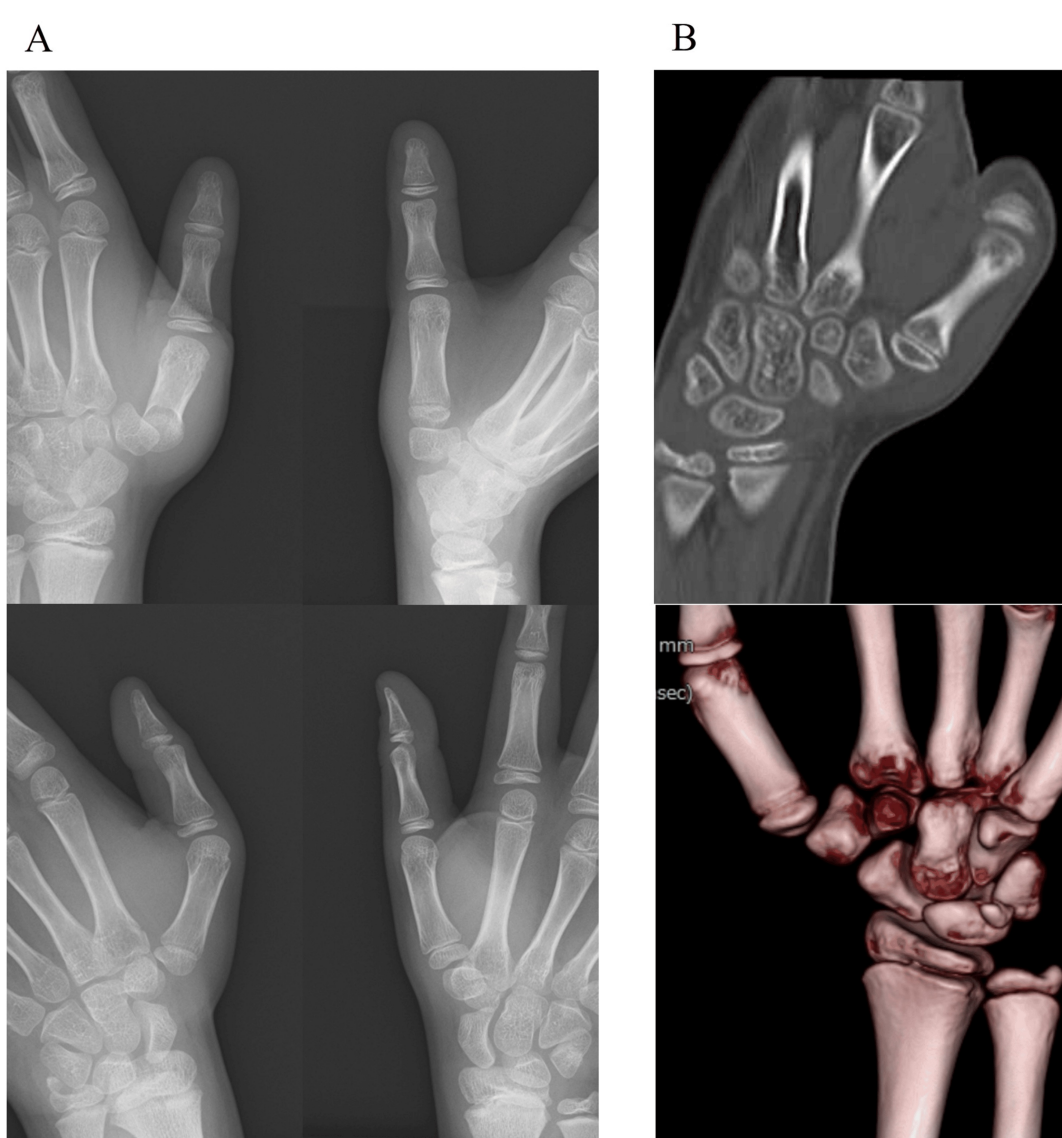
Images before the initial surgery. (A) Bilateral hand radiographs and (B) computed tomography images showing the dislocated left thumb carpometacarpal (CMC) joint without fractures.

**Figure 2:**
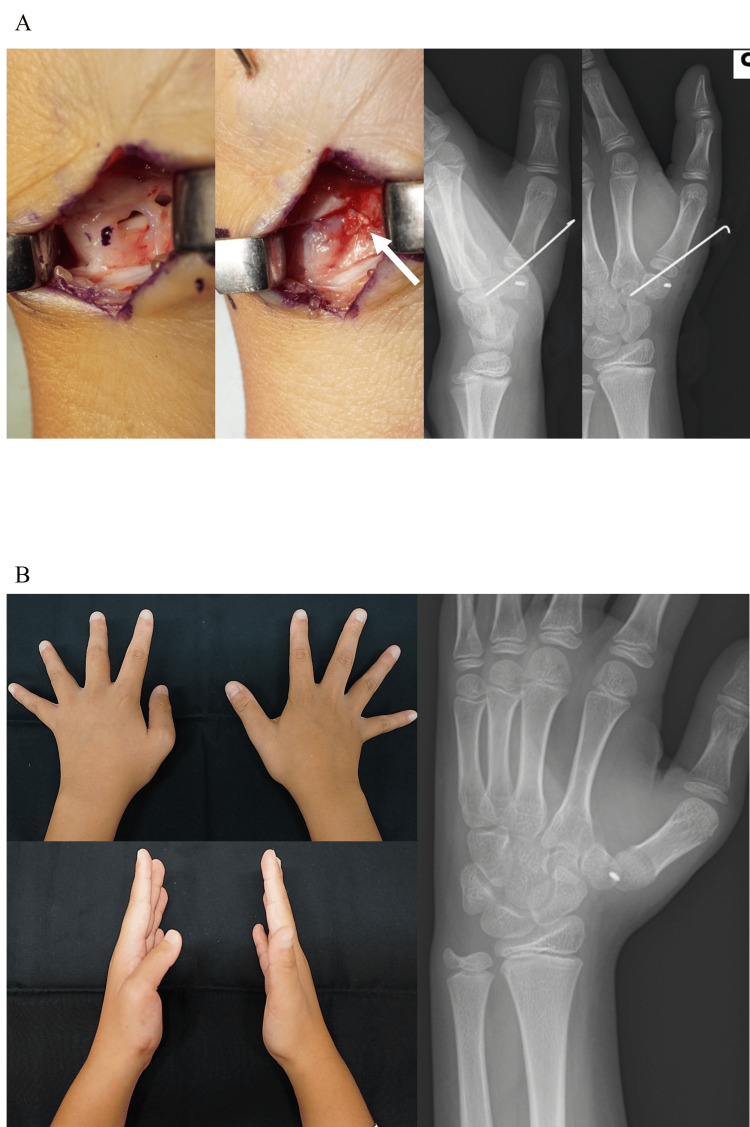
Images at and after the initial surgery. (A) Intraoperative finding of direct primary repair of the ligaments in the left thumb carpometacarpal (CMC) joint. The white arrow shows the sutured volar anterior oblique ligament. Postoperative radiograph showing the reduced CMC joint of the left thumb with percutaneous K-wire fixation. (B) Four months after the surgery: Gross appearance and the radiograph showing recurrent CMC joint dislocation of the left thumb.

**Figure 3:**
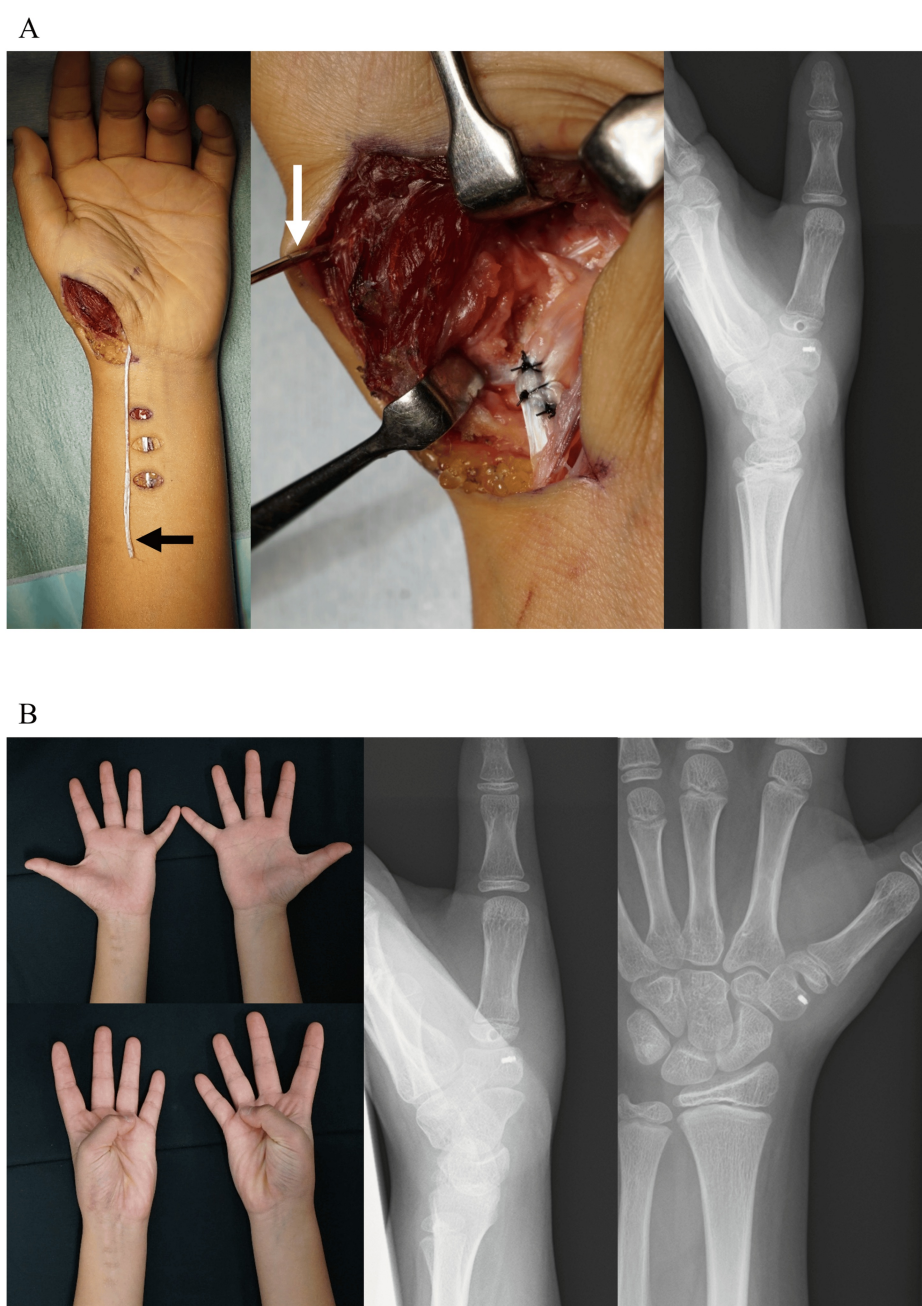
Images at and after the secondary surgery. (A) Intraoperative finding of secondary Eaton-Littler’s ligament reconstruction. The black arrow shows a half slip of the flexor carpi radialis tendon. The white arrow shows a guide wire for drilling from the volar to the dorsal region at the base of the first metacarpal while preserving the growth plate. A postoperative radiograph shows the reduced carpometacarpal (CMC) joint of the left thumb with internal stabilization using a half-slip of the flexor carpi radialis. (B) Fourteen months after secondary ligament reconstruction surgery. Full thumb range of motion of the left hand compared with the right hand. The left thumb CMC joint is completely stable in the reduced position on X-ray images.

A healthy 11-year-old boy, who belonged to a junior soccer club team, sustained an injury to his left thumb by pulling on it during soccer. He was referred to our hand clinic 10 days after the injury. His left thumb was deformed with flexion in the metacarpophalangeal joint and pain. General examination showed systematic hyperjoint laxity, with a Beighton score of 8 [10]. The thumb CMC joint was dislocated dorsally and radially without fractures on X-ray and computed tomography images (Figure 1). The dislocated thumb CMC joint was easily reducible under brachial plexus anesthesia, but could not be maintained in the reduced position. Thus, open reduction and direct primary repair of ligaments in the thumb CMC joint were performed on the day of consultation. Through a mid-lateral incision of the thumb CMC joint, the dorsoradial ligament (DRL), which had been detached dorsally from the trapezium, was repaired with a suture anchor, and the volar anterior oblique ligament (AOL), which had torn in the middle part, was directly sutured between their remnants (Figure 2A). Hereby, the dislocated thumb CMC joint could be maintained in the reduced position. Temporary pinning of the thumb CMC joint and thumb spica cast was applied for three weeks. Three months after the surgery, the thumb CMC joint was stable and painless. However, the thumb CMC joint was dislocated again and painful with its deformity four months after the surgery, when a heavy package fell onto his left thumb (Figure 2B). As the left thumb CMC joint was recurrently dislocated on the X-ray image, open reduction and secondary Eaton-Littler’s ligament reconstruction were performed [11]. Through a Wagner’s incision, the radial half of the flexor carpi radialis (FCR) tendon was harvested from its musculotendinous junction to its insertion with the left attached to the trapezium and used to reconstruct both the DRL and AOL, as described previously (Figure 3A) [11]. A 1.8 mm drill hole was made using a cannulated drill (Acutrack 2 Micro; Acumed LLC, Hillsboro, OR) through a 0.9 mm guide wire in the proximal metacarpal, distal to the physis and parallel to it in a plane perpendicular to the axis of the thumbnail, taking extreme caution to avoid injury to the growth plate under intraoperative fluoroscopy. The tendon was passed through the drill hole in a volar to dorsal orientation and then brought over the remaining FCR and tightly sutured to itself. For thumb CMC stabilization, a thumb spica cast was applied for seven weeks, and a thumb brace for the subsequent four months. Fourteen months after the surgery, the thumb CMC joint was stable in the reduced position and painless without limitation of the range of motion of his left thumb (Figure 3B). Grip strength was 19.4 kg on the right and 18.2 kg on the left. Tip and key pinch strength were 3.5 and 5.5 kg on the right and 3.0 and 5.0 kg on the left, respectively. Quick-Disabilities of the Arm, Shoulder, and Hand and Hand20 scores were 2.3 and 5, respectively, and he could return to his original midfielder position.

## Discussion

Traumatic dislocation of the thumb CMC joint accounts for less than 1% of all hand injuries and is even less common in children [1,8]. Only eight pediatric patients between 8 and 14 years of age with thumb CMC traumatic isolated dislocations without fractures have been reported in the English literature [3-9]. Four of these eight patients were successfully treated with closed reduction with casting or percutaneous pinning, while the other four patients failed to maintain reduction of the thumb CMC joint after the initial treatment of closed reduction with casting. Three of the four pediatric patients who failed to maintain reduction of the thumb CMC joint eventually needed additional ligament reconstruction [5,8,9]. In adult thumb CMC dislocation, as closed reduction and percutaneous pinning have been considered to be inadequate and associated with risks of recurrent subluxation, open reduction with capsular ligament repair is usually indicated [12]. However, in children, open surgery with ligament repair remains controversial, as described above [8]. In particular, there have been no reports of postoperative recurrence of thumb CMC dislocation, as in the present case, despite performing initial surgical treatment with open reduction and direct ligament repairs in pediatric thumb CMC dislocations.

There are several possible reasons for the recurrence of thumb CMC joint dislocation in the present case. First, he had generalized hyperjoint laxity, including the thumb, which was evidenced by a Beighton score of 8 [10]. This was a very common type of idiopathic joint hypermobility, not joint hypermobility syndrome as Ehlers-Danlos syndrome, Marfan syndrome, and Down syndrome. Second, the duration of cast immobilization with temporary pinning after the initial surgery was as short as three weeks, despite having generalized joint laxity. Third, the excessive direct axial compression load was simply directed at the thumb in the second traumatic event, leading to the re-rupture of the repaired ligament. Thus, recurrent thumb CMC joint dislocation may have been caused by these multiple complex factors.

## Conclusions

We report the first case of an 11-year-old boy with recurrent left thumb CMC joint dislocation due to idiopathic generalized hyperjoint laxity, even after primary open reduction with capsular ligament repair of the thumb CMC joint, eventually treated with Eaton-Littler’s ligament reconstruction. Although acute traumatic dislocation without fractures of the thumb CMC joint is extremely rare in children and treatment options remain controversial, primary ligament reconstruction of the thumb CMC joint may be considered in pediatric patients with systemic joint laxity or recurrent thumb CMC joint dislocation. Eaton-Littler’s ligament reconstruction is recommended for thumb CMC joint stability because two prime stabilizers of the DRL and AOL are appropriately reconstructed by a half-slip of the FCR tendon in this procedure.
